# An algal enzyme required for biosynthesis of the most abundant marine carotenoids

**DOI:** 10.1126/sciadv.aaw9183

**Published:** 2020-03-04

**Authors:** O. Dautermann, D. Lyska, J. Andersen-Ranberg, M. Becker, J. Fröhlich-Nowoisky, H. Gartmann, L. C. Krämer, K. Mayr, D. Pieper, L. M. Rij, H. M.-L. Wipf, K. K. Niyogi, M. Lohr

**Affiliations:** 1 Institut für Molekulare Physiologie, Pflanzenbiochemie, Johannes Gutenberg-Universität, 55128 Mainz, Germany.; 2 Molecular Biophysics and Integrated Bioimaging Division, Lawrence Berkeley National Laboratory, Berkeley, CA 94720, USA.; 3 Department of Plant and Microbial Biology, University of California, Berkeley, Berkeley, CA 94720, USA.; 4 Howard Hughes Medical Institute, University of California, Berkeley, Berkeley, CA 94720, USA.

## Abstract

Fucoxanthin and its derivatives are the main light-harvesting pigments in the photosynthetic apparatus of many chromalveolate algae and represent the most abundant carotenoids in the world’s oceans, thus being major facilitators of marine primary production. A central step in fucoxanthin biosynthesis that has been elusive so far is the conversion of violaxanthin to neoxanthin. Here, we show that in chromalveolates, this reaction is catalyzed by violaxanthin de-epoxidase–like (VDL) proteins and that VDL is also involved in the formation of other light-harvesting carotenoids such as peridinin or vaucheriaxanthin. VDL is closely related to the photoprotective enzyme violaxanthin de-epoxidase that operates in plants and most algae, revealing that in major phyla of marine algae, an ancient gene duplication triggered the evolution of carotenoid functions beyond photoprotection toward light harvesting.

## Teaser

A key enzyme in light-harvesting pigment formation in many algae stems from duplication of a gene involved in photoprotection.

## INTRODUCTION

Chromalveolate algae, in particular heterokonts, haptophytes, and dinophytes, are major contributors to marine primary production and global carbon fixation (*1*–*3*). The brown color displayed by most of these algae is caused by the presence of carotenoids such as fucoxanthin, 19′-hexanoyloxyfucoxanthin, or peridinin (*4*), which efficiently collect green light (*5*). These pigments are limited to chromalveolates, and their contribution to photosynthetic light harvesting is equal to or even higher than that of the accessory pigment chlorophyll *b* in the light-harvesting complexes of land plants and green algae (*5*, *6*). Carotenoids are also important for protecting the photosynthetic machinery against damage by excessive light intensities. As constitutive photoprotective defense, they participate in quenching of triplet chlorophyll and singlet oxygen (*7*). Most eukaryotic phototrophs also have an inducible carotenoid-based photoprotective mechanism that involves a xanthophyll cycle. While land plants, green algae, and some groups of chromalveolate algae use the violaxanthin cycle, most chromalveolates use the diadinoxanthin cycle instead (*8*).

As haptophytes and heterokonts, particularly diatoms, are major constituents of many marine phytoplankton communities, fucoxanthin and its derivatives such as 19′-hexanoyloxyfucoxanthin and 4-keto-19′-hexanoyloxyfucoxanthin present in many haptophyte algae (*9*), and 19′-butanoyloxyfucoxanthin in some haptophytes and also in pelagophyte algae (*4*), are the most abundant carotenoids in the world’s oceans (*2*, *10*). A characteristic structural feature uniting these carotenoids is the presence of an allenic group consisting of two cumulated double bonds (*11*), and current evidence suggests the allenic carotenoid neoxanthin as the central intermediate in their biosynthesis (*11*–*14*). Carotenoid biosynthesis in diatoms such as *Phaeodactylum tricornutum* has been studied by physiological experiments (*15*, *16*), and several of the involved genes have been identified by comparative genomics (*17*, *18*) and heterologous expression (*12*, *19*). As diatoms are diploid, a search for novel carotenogenic genes by unbiased forward genetic screens such as random mutagenesis is not feasible (*20*). Other heterokont algae, such as the eustigmatophytes *Nannochloropsis oceanica* or *Nannochloropsis gaditana* are more suitable for such approaches because they are haploid, and complete genome sequences and advanced genetic tools are available for these organisms (*21*).

The major allenic carotenoid species in eustigmatophytes are acyl esters of vaucheriaxanthin, which is presumably synthesized from the same biosynthetic precursors as fucoxanthin (*11*, *13*). However, the amount of vaucheriaxanthin esters and their contribution to light harvesting in eustigmatophyte algae is limited (*22*, *23*), suggesting that the loss of these carotenoids will have less marked consequences than the loss of fucoxanthin for diatoms. We therefore used the eustigmatophyte alga *N. oceanica* to generate mutants by random insertional mutagenesis and screened the resulting mutant library for clones with altered chlorophyll fluorescence properties, indicating an altered composition of photosynthetic pigments (*24*, *25*). Here, we report on the identification and characterization of a *N. oceanica* pigment mutant defective in a key gene of vaucheriaxanthin biosynthesis that is conserved in chromalveolate algae. By cloning of the corresponding gene from *N. oceanica* and eight other chromalveolate algae and functional characterization of the expression products, we demonstrate that this gene is also central to the formation of other allenic carotenoids such as fucoxanthin and peridinin.

## RESULTS AND DISCUSSION

### An algal mutant devoid of allenic carotenoids is defective in VDL

**Fig. 1:**
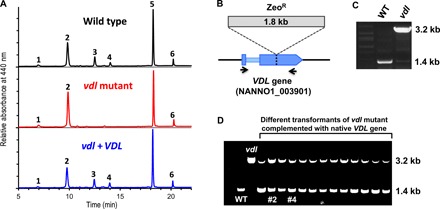
A *vdl* knockout mutant of *N. oceanica* no longer synthesizes the allenic vaucheriaxanthin acyl esters. (**A**) HPLC analyses (system IIb) of pigment extracts from wild type, the *vdl* mutant, and a *vdl* mutant complemented with its native *VDL* gene (*vdl* + *VDL*, clone #4) demonstrate that knockout of the *VDL* gene results in loss of vaucheriaxanthin acyl esters (peaks 3 and 4) and a concomitant increase in violaxanthin (2). Chromatograms were normalized to chlorophyll *a* (5); other peaks were identified as latoxanthin (1) and β-carotene (6). For detailed pigment stoichiometries in the wild type, the *vdl* mutant, and two *VDL*-complemented strains of the *vdl* mutant, see table S1. (**B**) Scheme showing the insertion site of the 1.8-kb zeocin resistance cassette (Zeo^R^) within the second exon of the *VDL* gene in the *vdl* mutant. Binding sites of the primers used for differentiation of wild-type (WT) and mutated *VDL* gene are indicated by black arrows. (**C**) Agarose gel showing 1.8-kb size difference of polymerase chain reaction (PCR) products of WT or the VDL-deficient mutant (*vdl*) using PCR primers specified in (B). (**D**) Agarose gel showing additional band of WT *VDL* fragment at 1.4 kb for PCR products from successfully complemented transformants of the *vdl* mutant; clones #2 and #4 were used for pigment analyses. Other experimental details are described in Materials and Methods.

By colony screening of a random-insertion mutant library of the eustigmatophyte alga *N. oceanica* for altered chlorophyll fluorescence using video imaging (*24*), we successfully identified a mutant completely devoid of vaucheriaxanthin esters (Fig. 1A). The lack of vaucheriaxanthin esters was accompanied by a compensatory increase in violaxanthin from about 300 mmol/mol Chl *a* in the wild type to about 440 mmol/mol Chl *a* in the mutant (table S1). Moreover, the mutant showed a slight but significant increase in a minor pigment that we tentatively identified as latoxanthin (fig. S1) and in the xanthophyll cycle pigments antheraxanthin and zeaxanthin (table S1), indicating a slightly increased xanthophyll cycle activity in the mutant under the applied growth conditions.

The lack of allenic carotenoids and the increased violaxanthin content suggested that the mutation may specifically affect the conversion of violaxanthin to neoxanthin, the first intermediate in the vaucheriaxanthin biosynthetic pathway. Mapping of the insertion site of the resistance cassette in the genome of the *N. oceanica* mutant revealed that it had disrupted a gene encoding a violaxanthin de-epoxidase–like (VDL) protein (Fig. 1, B and C). VDL is closely related to violaxanthin de-epoxidase (VDE) that operates in the photoprotective xanthophyll cycle of plants and most algae and converts violaxanthin to zeaxanthin (*17*), but so far, no function has been assigned to VDL. Transformation of the mutant with its native *VDL* gene (Fig. 1D) restored the wild-type pigment phenotype (Fig. 1A), confirming that the VDL protein is essential for the biosynthesis of allenic carotenoids in this alga. Detailed pigment analyses of two different complemented mutant lines (table S1, clones *vdl + VDL* #2 and #4) revealed that both accumulated the same two vaucheriaxanthin esters as the wild type, although with slightly different molar ratios to Chl *a*. Both lines also had a moderately increased zeaxanthin content, again indicating a minor xanthophyll cycle activity under the applied growth conditions. The sum of all xanthophylls (violaxanthin, antheraxanthin, zeaxanthin, latoxanthin, and the two vaucheriaxanthin esters) per Chl *a*, however, was not significantly different between the wild type and any of the three mutant lines (table S1).

### VDL is limited to chromalveolate algae and predicted to reside inside the plastid thylakoids

VDL is ubiquitous in chromalveolates but absent from land plants and green algae (*17*). To substantiate the general importance of VDL for the formation of allenic carotenoids in chromalveolates, we searched the genomes or transcriptomes of 18 chromalveolate species for *VDL* genes. On the basis of these searches, we cloned *VDL* genes from *N. oceanica* and eight other algae covering the three major chromalveolate clades Heterokontophyta (Stramenopiles), Haptophyta, and Dinophyta and functionally characterized their expression products. A particular emphasis was put on the diatom *P. tricornutum* as the best-studied model organism for the biosynthesis of fucoxanthin. In line with previous results (*17*), we found *VDL* genes in all 18 chromalveolate species we examined, with diatoms and haptophytes having two *VDL* genes each (fig. S2). In silico analyses of the deduced protein sequences predicted that VDL is localized within the thylakoid lumen of plastids (data file S1). In chromalveolate algae, targeting of nucleus-encoded proteins to the thylakoid lumen is facilitated by a tripartite presequence at their N terminus consisting of a signal peptide and a chloroplast transit peptide followed by a second signal peptide (*25*). Analyses of the VDL sequences using multiple targeting prediction tools identified N-terminal presequences with all three targeting elements in 15 of 18 VDL proteins (data file S1). Notably, the second signal peptide necessary for transport from the plastid stroma into the thylakoid lumen was detected in all 18 presequences. VDL is closely related to VDE, which is known to be a luminal protein (*8*). Therefore, we validated the predictions for VDL by also analyzing the VDE sequences from 17 chromalveolates and 4 green plants/algae, with the expected targeting elements for luminal localization predicted in all investigated proteins (data file S1).

### Expression of VDL in tobacco induces the accumulation of *trans*-neoxanthin

**Fig. 2:**
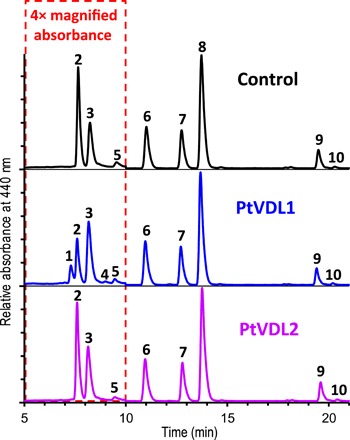
Transient expression of *VDL1* from *P. tricornutum* in tobacco leaves results in accumulation of neoxanthin. HPLC analysis (system Ib) of pigment extracts from untreated leaves (control) and leaves after *Agrobacterium*-mediated transformation with *VDL1* or *VDL2* from *P. tricornutum*. Chromatograms were normalized to chlorophyll *a* (peak 8), and the parts showing neoxanthin (1), violaxanthin (2), 9′-*cis*-neoxanthin (3), deepoxyneoxanthin (4), and antheraxanthin (5) were magnified four times. Other peaks were identified as lutein (6), chlorophyll *b* (7), β-carotene (9), and 9-*cis*-β-carotene (10). For detailed pigment stoichiometries in leaf samples from the three treatments, see table S2.

For functional characterization of VDL, we first used *Agrobacterium*-mediated transient expression of *VDL* genes from nine different chromalveolate algae in leaves of tobacco (*Nicotiana benthamiana*) (*26*, *27*). To ensure luminal targeting of the algal VDL proteins in the tobacco plastids, we replaced their native targeting sequences by the targeting sequence of the lumen-localized VDE from tobacco (*Nicotiana tabacum*). Transformation of tobacco leaves with the accordingly modified *VDL1* gene from *P. tricornutum* (*PtVDL1*) resulted in the accumulation of the *trans*-isomer of neoxanthin (hereafter referred to simply as neoxanthin) to about 14 mmol/mol Chl *a* and a reduction in the violaxanthin content by about 18 mmol/mol Chl *a* (Fig. 2 and table S2). Targeting of PtVDL1 to the plastid stroma by the transit sequence of the zeaxanthin epoxidase from *Arabidopsis thaliana* (*27*) led to a much lower accumulation of neoxanthin in tobacco leaves compared with luminal targeting (table S2), indicating a higher activity of the enzyme at the more acidic pH in the thylakoid lumen. In leaves transformed with *PtVDL1*, the concentration of 9′-*cis*-neoxanthin also increased significantly by about 26 mmol/mol Chl *a* for lumen-targeted PtVDL1 (*P* = 2 × 10^−7^) and by about 16 mmol/mol Chl *a* for stroma-targeted PtVDL1 (*P* = 3 × 10^−6^), suggesting that part of the neoxanthin generated by PtVDL1 may have been converted to 9′-*cis*-neoxanthin by an endogenous enzyme (Fig. 2 and table S2). In land plants, however, the exact route and the enzymes involved in the formation of 9′-*cis*-neoxanthin from violaxanthin are still unknown (*28*). Transformation of tobacco leaves with the *VDL2* gene from *P. tricornutum* (*PtVDL2*) fused to a luminal targeting sequence did not result in the accumulation of neoxanthin or any other additional carotenoid (Fig. 2 and table S2), suggesting that VDL2 is not involved in the biosynthesis of neoxanthin in *P. tricornutum* and other chromalveolate algae.

**Fig. 3:**
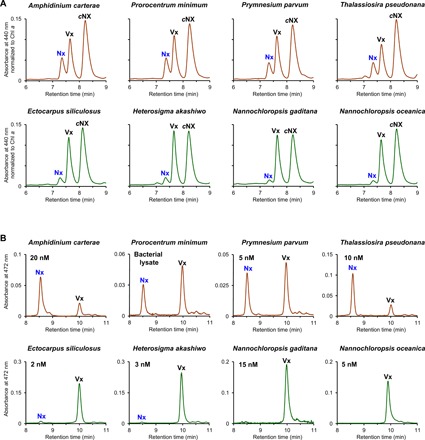
Violaxanthin-neoxanthin tautomerase activity of VDL proteins from further chromalveolate algae in tobacco leaves and in vitro. (**A**) HPLC analyses (system Ib) of pigment extracts from tobacco leaves transiently expressing VDL from algae with diadinoxanthin cycle (brown-colored chromatograms) or from algae that use the violaxanthin cycle for photoprotection (green-colored chromatograms). Depicted are details of chromatograms normalized to the chlorophyll *a* peak showing *trans*-neoxanthin (Nx), violaxanthin (Vx), and 9′-*cis*-neoxanthin (cNx). Blue label indicates that *trans*-neoxanthin is only found in leaves transformed with the algal *VDL* genes. Note the stronger accumulation of *trans*-neoxanthin when leaves are transformed with *VDL* from algae with diadinoxanthin cycle. Detailed pigment stoichiometries in leaf samples from all treatments are given in table S3. (**B**) HPLC analyses (system II) of samples from in vitro assays with recombinant algal VDL proteins using violaxanthin as substrate and incubation times of 3 hours [chromatogram colors and peak labels as in (A), reaction products labeled blue; values below species names indicate VDL concentrations in assay; activity of VDL from *P. minimum* was measured using bacterial lysate]. For each VDL, at least two different preparations were examined with similar results. VDL concentrations in the assays varied because of different expression efficiencies of VDL proteins in *E. coli* that could not be overcome by changing expression conditions. VDL proteins from algae with diadinoxanthin cycle showed substantial in vitro activities that were correlated with protein concentrations in the assays, while VDL proteins from algae with violaxanthin cycle showed minor or no activity independent of the protein concentrations used. Other experimental details are described in Materials and Methods.

To substantiate the general importance of VDL proteins in chromalveolate carotenoid biosynthesis, we additionally performed the transient expression of lumen-targeted VDL from five other heterokont algae (the diatom *Thalassiosira pseudonana*, the eustigmatophytes *N. oceanica* and *N. gaditana*, the brown alga *Ectocarpus siliculosus*, and the raphidophyte *Heterosigma akashiwo*), two dinophytes (*Amphidinium carterae* and *Prorocentrum minimum*), and a haptophyte (*Prymnesium parvum*) in tobacco leaves. In all cases, expression of the algal *VDL* gene induced the accumulation of neoxanthin in parallel with a decrease in violaxanthin (Fig. 3A and table S3), further supporting the conclusion from the VDL-defective mutant of *N. oceanica* that VDL catalyzes the conversion of violaxanthin to neoxanthin in chromalveolates.

### In vitro characterization of VDL1 from the diatom *P. tricornutum* confirms violaxanthin as main substrate

**Fig. 4:**
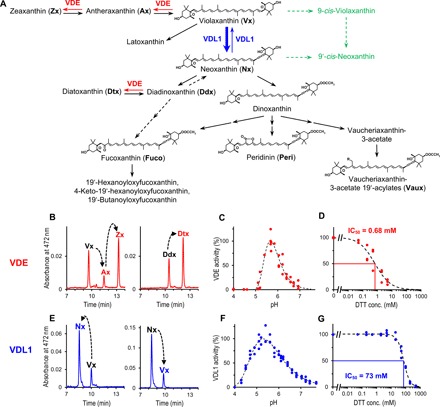
Functional comparison of VDE and VDL1 from *P. tricornutum*. (**A**) Proposed pathway of carotenoid biosynthesis in chromalveolate algae and reactions catalyzed by VDE (red arrows) and VDL (blue arrows). In addition, the two putative pathways to 9′-*cis*-neoxanthin—a neoxanthin isomer specific to land plants and green algae—are indicated by broken arrows (green-colored paths; enzymes unknown). (**B** to **D**) HPLC analyses (system II) of in vitro assays with recombinant VDE showing (B) enzymatic de-epoxidation of Vx to Ax and Zx and of Ddx to Dtx (reaction products labeled red); (C) pH dependence of VDE activity; and (D) dose-response curve of VDE inhibition by reductant DTT. (**E** to **G**) In vitro assays with recombinant VDL1 showing (E) enzymatic tautomerization of Vx to Nx and of Nx to Vx (reaction products labeled blue); (F) pH dependence of VDL1 activity; and (G) dose-response curve of VDL1 inhibition by DTT. The experimental details are described in Materials and Methods.

The close relation of VDL with VDE prompted us to compare the properties of both enzymes in more detail. The lumen-localized VDE is a key enzyme of the photoprotective violaxanthin cycle in higher plants and green algae. Under high light-induced (over)acidification of the lumen, VDE is activated and catalyzes the elimination of the two epoxy groups in violaxanthin to yield zeaxanthin via the intermediate antheraxanthin (Fig. 4A). Zeaxanthin supports the dissipative deactivation of singlet excited chlorophyll in the light-harvesting antennae in a process called energy-dependent quenching (qE) (*29*, *30*). Many chromalveolate algae use the diadinoxanthin cycle instead. Here, VDE converts the monoepoxide diadinoxanthin to diatoxanthin (Fig. 4A), which supports qE in a fashion similar to zeaxanthin in the violaxanthin cycle (*8*). Chemosystematic considerations and experimental data are in strong support of diadinoxanthin also being synthesized via violaxanthin and neoxanthin as obligatory intermediates (*12*–*16*).

For a more detailed comparison of chromalveolate VDL with VDE, we chose the genes *PtVDE*, *PtVDL1*, and *PtVDL2* from *P. tricornutum*. After heterologous expression of the three genes in *Escherichia coli*, the recombinant proteins were purified, refolded, and subjected to an in vitro assay commonly used for measuring VDE activity (*31*, *32*). Using either violaxanthin or diadinoxanthin as substrate, PtVDE showed the expected de-epoxidase activity by converting both violaxanthin to zeaxanthin and diadinoxanthin to diatoxanthin (Fig. 4B). In line with the results from transient expression in tobacco leaves, PtVDL2 showed no catalytic activity (fig. S3), whereas PtVDL1 converted violaxanthin to neoxanthin (Fig. 4E). However, the conversion was incomplete, terminating at a ratio of 75% neoxanthin to 25% violaxanthin. When PtVDL1 was offered neoxanthin as substrate, slightly more than 20% was converted to violaxanthin (Fig. 4E), demonstrating that PtVDL1 catalyzed the epoxy-enol tautomerization of violaxanthin and neoxanthin with an equilibrium ratio of about 25:75.

PtVDL1 was also able to isomerize the monoepoxide antheraxanthin to the allenic deepoxyneoxanthin and vice versa (fig. S4, B and C), albeit with a much slower rate (fig. S5). Other epoxy xanthophylls such as diadinoxanthin and 9′-*cis*-neoxanthin—the major neoxanthin isomer in land plants—were not substrates for PtVDL1 (fig. S4, D and F). Notably, dinoxanthin (neoxanthin 3-acetate), which is a putative intermediate in the biosynthesis of fucoxanthin, peridinin, and vaucheriaxanthin esters (Fig. 4A), also was not a substrate of PtVDL1 (fig. S4E). Acetylation of the hydroxyl group at C3 of neoxanthin apparently makes the enzymatic allene formation irreversible and thus may represent the committed step in the biosynthesis of allenic light-harvesting carotenoids in chromalveolates.

Together, these findings suggest that violaxanthin is the major native substrate of PtVDL1 and support the earlier proposal (*11*) that in algae using the diadinoxanthin cycle, not only the xanthophyll cycle–dependent formation of diatoxanthin but also its de novo biosynthesis under high light stress occurs mainly via de-epoxidation of diadinoxanthin by VDE. However, the observation that tobacco leaves expressing chromalveolate VDLs accumulated not only neoxanthin but also small amounts of deepoxyneoxanthin (Fig. 2 and tables S2 and S3) indicates that diatoxanthin may, to some extent, also be formed directly from antheraxanthin via deepoxyneoxanthin (see fig. S4), which could be an explanation for the previously reported increase of diatoxanthin in the diatom *P. tricornutum* under very high light despite the presence of a VDE inhibitor (*33*, *34*). Moreover, our data suggest that, contrary to earlier speculations (*17*, *35*), neither VDL1 nor VDL2 is involved in the de-epoxidation of violaxanthin and diadinoxanthin in *P. tricornutum* and other chromalveolate algae.

### VDL differs from VDE in cofactor requirement, pH range, and sensitivity against reducing agents

The potential competition of VDL and VDE for violaxanthin suggests that mechanisms for differential regulation of their activities have evolved. As expected for luminal enzymes, the in vitro activities of both PtVDE and PtVDL1 (Fig. 4, C and F) showed optima between pH 5 and 6. At neutral pH, however, PtVDE activity was almost zero, while PtVDL1 had a residual activity of about 20%. The VDE from land plants is strictly dependent on the cofactor ascorbate for reductive elimination of the epoxy group and can be inhibited by reducing agents such as dithiothreitol (DTT) (*8*). Reducing agents target a number of highly conserved cysteine residues in the N-terminal domain of VDE that form disulfide bridges and have been implicated in redox-dependent regulation of VDE activity by thioredoxins (*36*, *37*). We found that the activity of PtVDE also was ascorbate dependent (fig. S6B) and was inhibited by DTT with a half maximal inhibitory concentration (IC_50_) of 0.68 mM (Fig. 4D), similar to the VDE from land plants (*38*). PtVDL1 activity, on the other hand, did not necessitate the addition of ascorbate (fig. S6A) or other cofactors in the in vitro assay, although the presence of ascorbate in the assay accelerated the reaction by about 10% (fig. S6A). Moreover, PtVDL1 was inhibited by DTT only at 100-fold higher concentrations than PtVDE (IC_50_ = 73 mM; Fig. 4G), although PtVDL1 contains six of the eight cysteine residues conserved in PtVDE (*17*). These results point to a constitutive activity of PtVDL1 modulated only by pH, whereas VDE activity is controlled on multiple levels, among them a pH-dependent affinity for its cofactor ascorbate (*39*) and the redox state in the thylakoid lumen (*36*, *37*).

### VDL activity is correlated with the type of xanthophyll cycle in the source alga

**Fig. 5:**
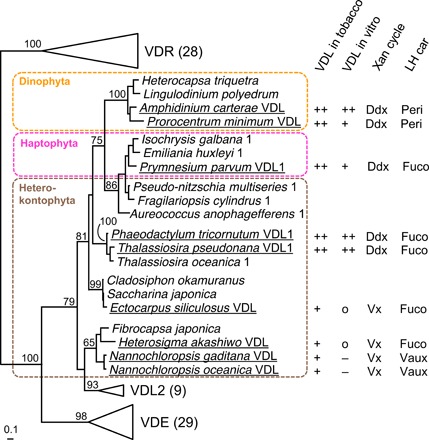
Phylogeny and summary of enzymatic activities in tobacco or in vitro of VDL proteins from selected dinophyte, haptophyte, and heterokont algae. Investigated VDL proteins are underlined, and their violaxanthin-neoxanthin tautomerase activities in tobacco or in vitro were indicated as strong (++), medium (+), weak (o), or not detectable (−) based on the results shown in Fig. 3. Also indicated are the type of xanthophyll cycle (Xan cycle) and the major allenic light-harvesting carotenoid (LH car) present in the respective algal species [for abbreviations of carotenoids, see Fig. 4]. Midpoint-rooted maximum likelihood tree of VDE family proteins from selected chromalveolate algae shows bootstrap supports (100 replicates) above 50% for major nodes. “VDL1” or “1” behind species name indicates additional presence of a VDL2 in that species. For VDE, VDL2, and VDR, values in brackets indicate the number of sequences included in the phylogenetic analysis. The full tree is shown in fig. S2.

Recombinant VDL1 proteins from the diatom *T. pseudonana*, the haptophyte *P. parvum*, and the dinophytes *A. carterae* and *P. minimum* also showed violaxanthin-neoxanthin tautomerase activity in vitro, while the VDL proteins from the four heterokonts *N. oceanica*, *N. gaditana*, *E. siliculosus*, and *H. akashiwo* failed to produce notable amounts of neoxanthin from violaxanthin under standard assay conditions (Figs. 3B and 5). Diatoms, haptophytes, and dinophytes use the diadinoxanthin cycle for photoprotection (Fig. 5) and usually contain only very low amounts of violaxanthin (*15*) (see also fig. S7A). The four algae whose VDL proteins were inactive in our assay, however, use the violaxanthin cycle (Fig. 5) and, thus, contain permanently high levels of violaxanthin (fig. S7B), which may necessitate a more stringent regulation of their VDL. In line with this suggestion, transformation of tobacco leaves with *VDL* genes from the four algae with the violaxanthin cycle resulted in a consistently lower accumulation of neoxanthin than with *VDL* genes from algae with diadinoxanthin cycle (Figs. 3A and 5 and table S3). In conclusion, the results suggest that the activity of VDL enzymes from algae with the violaxanthin cycle is fine-tuned by some yet unknown factor(s).

## CONCLUSIONS

As violaxanthin is a major substrate of both VDL and VDE, the two enzymes constitute a switch point for directing carotenoids either into the photoprotective or the light-harvesting branch of carotenoid biosynthesis in chromalveolate algae using the violaxanthin cycle for photoprotection (Fig. 4A). In chromalveolates using the diadinoxanthin cycle, the situation is more complex, as here neoxanthin is the precursor not only of the major light-harvesting carotenoids but also of diadinoxanthin (*12*, *13*). Here, the emergence of VDL additionally triggered the evolution of a novel xanthophyll cycle that appears to be superior to the violaxanthin cycle, as these algae contain negligible amounts of violaxanthin, antheraxanthin, and zeaxanthin under most growth conditions (*13*, *15*, *16*, *40*, *41*). Under exceptional conditions that promote high VDE activity in combination with substantial de novo synthesis of carotenoids, algae using the diadinoxanthin cycle may transiently accumulate also the pigments of the violaxanthin cycle (*15*). Recent experimental evidence, however, suggests that in these algae zeaxanthin is ineffective in mediating photoprotection and may even interfere with the photoprotective function of diatoxanthin (*42*, *43*).

The finding that VDL and VDE have arisen from a common ancestor by gene duplication (*17*) (Fig. 5) provides a notable example of how photoprotective and light-harvesting functions of carotenoids in chromalveolate algae coevolved at the molecular level. The lack of *VDL* genes in land plants, on the other hand, indicates that the biosynthesis of neoxanthin from violaxanthin in chromalveolate algae and that of 9′-*cis*-neoxanthin from violaxanthin in land plants and green algae evolved independently.

## MATERIALS AND METHODS

### Plant material and growth conditions

*P. tricornutum* (strain UTEX 646), *Mischococcus sphaerocephalus* [SAG (Sammlung von Algenkulturen) 874-1], *N. gaditana* (SAG 2.99), and *N. oceanica* (CCMP1779) were grown in modified ASP-2 medium (*16*). *T. pseudonana* (SAG 1020-1b) was grown in ½SWES + Na_2_SiO_3_ medium, *P. parvum* (SAG 127.79) in ½SWES medium, and *E. siliculosus* [Ec32 = CCAP (Culture Collection of Algae and Protozoa) 1310/4] in SWES medium (for media recipes, see SAG, Goettingen University, Germany; http://www.uni-goettingen.de/de/list-of-media-and-recipes/186449.html). *H. akashiwo* (CCMP2393), *A. carterae* (SAG 37.80), and *P. minimum* (CCMP1329) were grown in ASW medium (CCAP artificial seawater, Oban, UK; https://www.ccap.ac.uk/media/documents/ASW.pdf). Algae were grown as 200-ml batch cultures in 500-ml Erlenmeyer flasks at 18°C and a photosynthetically active photon flux density (PPFD) of 20 or (in case of *P. tricornutum*) 40 μmol·m^−2^·s^−1^ with a 16-hour light/8-hour dark cycle. Cultures of *P. tricornutum*, *M. sphaerocephalus*, *N. gaditana*, *N. oceanica*, and *T. pseudonana* were shaken at 120 rpm. *N. benthamiana* and *N. tabacum* were grown in soil pots on irrigation trays containing Hoagland’s nutrient solution at 22°C and a PPFD of 60 μmol·m^−2^·s^−1^ with a 15-hour light/9-hour dark cycle.

### Data mining and sequence analyses

Protein and/or nucleotide sequence data from chromalveolates available in the Centro di Ricerca Interdipartimentale per le Biotecnologie Innovative (CRIBI) Biotechnology Center Portal of the University of Padua (http://genomes.cribi.unipd.it/), GenBank (https://www.ncbi.nlm.nih.gov/), the Joint Genome Institute (JGI) Genome Portal (http://genome.jgi.doe.gov/), the Microbial Eukaryote Transcriptome Sequencing Project (*44*) (MMETSP; https://www.imicrobe.us/#/projects/104), and the Online Resource for Community Annotation of Eukaryotes (http://bioinformatics.psb.ugent.be/orcae/) were searched with the BLAST tool (*45*) using the amino acid sequences of the VDE family members from *P. tricornutum* (*17*) as input. MMETSP mining, protein alignments, and phylogenetic analyses using maximum likelihood methods with bootstrapping were performed as previously described (*27*). Accessions of sequence data used for cloning of algal genes and for in silico analyses are compiled in data file S2.

Prediction of N-terminal targeting signals and their putative cleavage sites in VDL and VDE protein sequences was done using TargetP 1.1 (*46*) (http://www.cbs.dtu.dk/services/TargetP/), SignalP 4.1 (*47*) (http://www.cbs.dtu.dk/services/SignalP/) and 3.0 (*48*) (http://www.cbs.dtu.dk/services/SignalP-3.0/), iPSORT (*49*) (http://ipsort.hgc.jp/), Predotar (*50*) (https://urgi.versailles.inra.fr/predotar/), HECTAR v1.3 (*51*) (http://webtools.sb-roscoff.fr/), PrediSi (*52*) (http://www.predisi.de/), and Signal-3L (*53*) (http://www.csbio.sjtu.edu.cn/bioinf/Signal-3L/). To further support signal peptide cleavage sites predicted by SignalP, protein sequences were manually searched for the presence of “ASAFAP” motifs (*54*). Then, sequences were shortened by the predicted signal peptide and analyzed again using TargetP, iPSORT, Predotar, and ChloroP 1.1 (*55*) (http://www.cbs.dtu.dk/services/ChloroP/) to identify putative chloroplast transit peptides. To identify the second signal peptide for import into the thylakoid lumen, all examined sequences were aligned and the beginning of the mature proteins was estimated from the conserved N-proximal regions in the alignment. Land plant VDE contains a Sec pathway–dependent signal peptide (*56*) ending with an A-X-A motif (*57*) for cleavage by the thylakoid processing peptidase. VDE and VDL sequences from algae were also found to contain A-X-A motifs, and sequence ranges covering 30 to 40 amino acids in front of these motifs were analyzed by TargetP, SignalP, iPSORT, Predotar, PrediSi, and Signal-3L.

### Cloning and expression of algal VDL and VDE

Total RNA from algae was prepared using either the InnuPREP Plant RNA Kit (Analytik Jena; used for *N. gaditana*, *P. parvum*, and *T. pseudonana*), the RNeasy Mini Kit (Qiagen; for *E. siliculosus* and *H. akashiwo*), the High Pure RNA Isolation Kit (Roche; for *A. carterae*), or TRIzol reagent (*58*) (Thermo Fisher Scientific; for *P. minimum* and *P. tricornutum*). Algal material (100 mg) was homogenized in liquid nitrogen by mortar and pestle (*N. gaditana*, *P. parvum*, *T. pseudonana*, and *E. siliculosus*) and then transferred to the kit-specific buffer or suspended directly in buffer and homogenized either in a Precellys Glass/Ceramic kit SK38/2-ml tube (Bertin Instruments) using a Mini-Beadbeater-1 (BioSpec) for 3 × 20 s at 5000 rpm (*H. akashiwo* and *A. carterae*), or by brief vortexing in a test tube containing micro glass beads of 0.2- to 0.25-μm diameter (*P. minimum* and *P. tricornutum*). Complementary DNA (cDNA) was synthesized from total RNA using the Transcriptor High Fidelity cDNA Synthesis Kit (Roche Life Science, Mannheim, Germany) and an anchored-oligo(dT)_18_ primer. Total RNA isolation of *N. oceanica* and cDNA synthesis was carried out as previously described (*26*). Cloning of genes and gene fragments for expression constructs was performed by polymerase chain reaction (PCR) using Phusion High-Fidelity DNA Polymerase (Thermo Scientific, Carlsbad, USA), with templates and gene-specific primers as listed in data file S3. Accuracy of cloned sequences was confirmed by comparison with reference sequences and with directly sequenced PCR products to ensure that deviations from reference sequences were caused by strain-specific single-nucleotide polymorphisms (see data file S4) and not by PCR errors.

Recombinant VDL and VDE proteins for in vitro assays were generated by heterologous expression in *E. coli* Rosetta (Novagen) using **pET-44a** (Novagen). Gene fragments encoding the mature proteins without targeting sequences were placed in-frame between the Nde I and Xho I sites of **pET-44a** by either conventional restriction and ligation or using In-Fusion Cloning HD (Takara) (see data file S3 for further details on PCR templates and primers, cloning strategies, and protein fragments encoded in the final expression constructs). Expression, preparation, and renaturation of the recombinant proteins were performed as previously described (*27*). Expression efficiency in *E. coli* and purity of the recombinant proteins in the inclusion body (IB) preparations were checked by SDS–polyacrylamide gel electrophoresis (*59*). The concentrations of recombinant protein in the in vitro assays were estimated from determination of the protein concentration in the respective IB preparation and calculation of the IB dilution in the renaturation buffer. Ten microliters of the tris-buffered IB suspension was diluted with 990 μl of A280-buffer (10 mM tris, pH 6.8; 2% SDS, w/v) and boiled for 2 min. Protein concentration was determined from the absorbance at 280 nm against A280 buffer, using extinction coefficient and molecular weight of the respective VDL protein sequence as calculated by the ExPASy ProtParam tool (http://web.expasy.org/protparam/).

Transient expression of algal *VDL* genes in tobacco leaves was achieved by *Agrobacterium*-mediated transformation of leaves of *N. benthamiana* using **pPZP200BAR**-based (*60*) expression constructs. For transient expression of *VDL* from *N. oceanica*, **pCAMBIA1300_35Su** (*61*) was used instead. Gene fragments encoding the mature proteins were placed in-frame behind a gene fragment encoding the N-terminal targeting sequence of the VDE from *N. tabacum* (tp_NtVDE_ = amino acid positions 1 to 134) for targeting of the expression products to the thylakoid lumen of tobacco plastids. In the case of the *VDL1* from *P. tricornutum*, the gene fragment was also placed behind a gene fragment encoding the targeting sequence of the zeaxanthin epoxidase from *A. thaliana* (tp_AtZEP_) for targeting of the protein to the plastid stroma as previously described (*27*). Further details on PCR templates and primers, cloning strategies, and protein fragments encoded in the final expression constructs are given in data file S3. For transient expression in *N. benthamiana*, leaves of the upper part of 6- to 8-week-old plants were infiltrated with *Agrobacterium* transformants harboring the respective expression constructs as previously described (*27*). Four days after infiltration, infiltrated and untreated control leaves were harvested, one to three leaf discs of 7-mm diameter were taken per leaf, immediately frozen in liquid nitrogen, lyophilized, and stored at −20°C until further analysis.

### In vitro assays with recombinant VDL and VDE

In vitro assays with recombinant VDL and VDE proteins under standard conditions (*31*, *32*) were carried out in reaction buffer at pH 5.2 containing 40 mM MES [2-(N-morpholino)ethanesulfonic acid], 10 mM KCl, and 5 mM MgCl_2_ and a final volume of 1 ml. Ten microliters of a 40 μM ethanolic stock solution of the respective carotenoid substrate and 10 μl of a 1.16 mM methanolic MGDG (monogalactosyl diacylglycerol; Lipid Products) stock were thoroughly mixed in a 1.5-ml reaction tube, followed by rapid addition of 920 μl of reaction buffer and vortexing for 10 s. For VDE assays and where explicitly stated for VDL assays, 20 μl of 1.5 M aqueous sodium ascorbate was added. The reaction was started by the addition of 60 μl of enzyme in renaturation buffer and vortexing for 10 s. Reaction tubes were incubated in a water bath at 20°C. Reactions were stopped by the addition of 300 μl of 1 M NaOH and incubation on ice. For determination of PtVDL1 reaction kinetics, assay volume was scaled up to 8 ml, and sample volume per time point was 1 ml. Pigment/lipid aggregates were harvested by centrifugation (18,000*g*, 2 min), the supernatant was removed, and the pellets were incubated at −20°C at least 15 min but not more than 24 hours until pigment extraction for high-performance liquid chromatography (HPLC) analysis.

For determination of the pH dependence of PtVDE and PtVDL1, the pH of the reaction buffer was adjusted with NaOH or HCl. Three independent experiments each covering the range of examined pH values were performed. PtVDL1 activity was estimated from the amount of violaxanthin converted to neoxanthin after 10 min, while PtVDE activity was determined from the amount of diadinoxanthin converted to diatoxanthin after 5 min. To account for minor activity differences between the three experiments, each dataset was fitted using an extreme value distribution function [*y* = a*e^(−*e*^[−((*x* − *b*)/*c*)] − ((*x* − *b*)/*c*) + 1); Eqn. 8033 in TableCurve 2D version 4; AISN Software], the maximum of the curve fit was set as 100%, and the measured activity data were normalized to this value. In Fig. 4 (C and F), the combined data were plotted against pH and a curve fit with Eqn. 8033 including all data points added.

For determination of DTT sensitivity, 5 M DTT in reaction buffer was added to the assays to attain final concentrations between 0.05 and 500 mM DTT. Enzymes were incubated with DTT for 10 min before reactions were started. Three independent experiments each covering the range of examined DTT concentrations including controls without DTT were performed. Enzyme activities were normalized to the activities of controls without DTT (=100%), and the combined activity data were plotted against DTT concentrations (Fig. 4, D and G). Dose-response curves of enzyme inhibition by DTT were calculated from the data using a three-parameter logistic function [*y* = *a*/(1 + (*x*/*b*)^*c*); Eqn. 8076 in TableCurve 2D].

For determination of the ascorbate dependence of PtVDL1 reaction kinetics, the assay volume was scaled to 5 ml. The assay was started by the addition of the enzyme already solubilized in buffer to the MGDG/violaxanthin solution. After vortexing for 5 s, the 5-ml assay was split immediately in two 2.5-ml aliquots that were pipetted to 50 μl of 1.5 M aqueous sodium ascorbate or 50 μl of demineralized water. At each time point, samples of 350-μl volume were withdrawn in parallel from the two aliquots, transferred to reaction vessels on ice and containing 100 μl of 1 N NaOH, and pigments were analyzed by HPLC as described above. Pigment analysis was performed by HPLC. The kinetics of violaxanthin decrease were fitted using an equilibrium concentration function [*y* = (*a* − *b*)*e^(−c*t) + *b*; Eqn. 8143 in TableCurve 2D]. For determination of the ascorbate dependence of PtVDE, an assay volume of 2 ml containing MGDG/violaxanthin and the enzyme solubilized in buffer was split in two 1-ml aliquots and either 20 μl of 1.5 M aqueous sodium ascorbate or 20 μl of demineralized water added. After 10 min, samples of 350-μl volume were withdrawn in parallel from the two aliquots and further processed as described for determination of the ascorbate dependence of PtVDL1.

### *Nannochloropsis* knockout and complementation

The *vdl* mutant of *N. oceanica* CCMP1779 was identified from a mutant library that was generated through random insertional mutagenesis with a zeocin-resistance cassette, in which the Sh *ble* gene had been fused to the *N. oceanica* β-tubulin promoter [1100 base pairs (bp) upstream of CCMP1779|8715-mRNA-1; https://genome.jgi.doe.gov/Nanoce1779/] (*62*) and RpL21 terminator (290 bp downstream of CCMP1779|9668-mRNA-1) by BglBricks cloning (*63*). The random insertion was mapped by SiteFinding PCR (*64*). For complementation, the *VDL* gene of *N. oceanica* was PCR amplified from cDNA with primers as specified in data file S3 and fused to the eIF3 promoter (926 bp upstream of CCMP1779|11214-mRNA-1) at the 5′ end and an Arf1 terminator at the 3′ end (496 bp downstream of CCMP1779|4318-mRNA-1) and to a hygromycin-resistance cassette (*62*) by Gibson cloning (New England Biolabs, Ipswich, USA). The linear fragment was transformed into the *vdl* mutant by electroporation as previously described (*65*). Transformants were selected on F2N medium (*65*) containing hygromycin B (50 μg/ml). Clones #2 and #4 were used for further analyses.

### Pigment extraction and analysis

Extraction of pigments from lyophilized tobacco leaf discs and lyophilized algal material was carried out as previously described (*27*). Algal material for pigment analysis was harvested from cultures 7 days after inoculation. Frozen pellets from the in vitro assays were extracted by the addition of 150 μl of extraction medium [81.1% methanol (v/v), 10.8% ethyl acetate (v/v), 8.1% water (v/v), 180 mM ammonium acetate]. Pigment extracts were analyzed on a Waters Alliance 2795 Separation Module equipped with a Waters 2996 photodiode array detector (Waters). Pigment extracts from tobacco leaf discs were analyzed using HPLC system I with a ProntoSIL 200-5 C30, 5.0 μm, 250 mm by 4.6 mm column equipped with a ProntoSIL 200-5-C30, 5.0 μm, 20 mm by 4.0 mm guard column (Bischoff Analysentechnik) and gradient conditions as previously described (*66*). For some measurements, system I was modified by using 85% instead of 90% acetonitrile as eluent B (HPLC system Ib), resulting in improved separation of *trans*-neoxanthin from violaxanthin. Pigment extracts from in vitro assays were analyzed on an HPLC system II with an EC 250/4 NUCLEOSIL 300-5 C18 column equipped with a CC 8/4 NUCLEOSIL 300-5 C18 guard column (Macherey-Nagel) and gradient conditions as previously described (*27*). For analysis of algal pigment extracts, the separation time of system II was increased by flattening the ternary gradient after 13 min, changing it to 30% B and 70% C at 16 min, 30% B and 70% C at 23 min, 100% B at 24 min, and 60% A and 40% B at 25 min (HPLC system IIb). Pigments were identified by comparison of retention times and absorbance spectra with those of reference pigments from a local pigment library and quantified as previously described (*13*). The identity of epoxy carotenoids was further confirmed by acid-catalyzed furanoid rearrangement of their 5,6-epoxy groups (*67*).

For identification of latoxanthin from *N. oceanica*, the carotenoid was isolated from a pigment extract of 400 ml of *N. oceanica* culture, dissolved in ethanol, and analyzed by circular dichroism (CD) spectroscopy (Jasco J-810, Jasco). The resulting spectrum (fig. S1A) was virtually identical with a previously published CD spectrum of latoxanthin from *Rosa foetida* (*68*). The presence of a 5,6-epoxy group was confirmed by treatment of the pigment with dilute HCl resulting in the expected 20-nm hypsochromic shift of the absorbance maxima (fig. S1B) (*67*). Because of its epoxy group, latoxanthin is more polar than the otherwise structurally identical heteroxanthin (fig. S1C). In agreement with this feature, we found the pigment from *N. oceanica* to elute 1 min earlier than heteroxanthin from the xanthophyte alga *M. sphaerocephalus* (*13*) on HPLC system IIb (fig. S1C). Latoxanthin has been tentatively identified before as a minor pigment in several other heterokont algae of the class Phaeophyceae (brown algae) (*69*), further corroborating the presence of latoxanthin in *Nannochloropsis* species.

As substrates for in vitro assays, antheraxanthin and violaxanthin were isolated from spinach (*Spinacia oleracea*), deepoxyneoxanthin from petals of *Lamium montanum* (*70*), diadinoxanthin from *P. tricornutum*, dinoxanthin from *A. carterae* (*71*), and *trans*-neoxanthin from *Mesostigma viride* (*72*).

### Statistical analysis

Differences in pigment stoichiometries between differently treated samples were analyzed for statistical significance using an unpaired two-tailed Student’s *t* test, and *P* values <0.05 were specified in the respective tables.

## Supplementary Material

http://advances.sciencemag.org/cgi/content/full/6/10/eaaw9183/DC1

Download PDF

Data file S1

Data file S2

Data file S3

Data file S4

An algal enzyme required for biosynthesis of the most abundant marine carotenoids

## SUPPLEMENTARY MATERIALS

Supplementary material for this article is available at http://advances.sciencemag.org/cgi/content/full/6/10/eaaw9183/DC1 Fig. S1. Tentative identification of latoxanthin from *N. oceanica*. Fig. S2. Midpoint-rooted maximum likelihood tree of VDE family proteins from selected species of chromalveolate algae and Viridiplantae (land plants and green algae). Fig. S3. In vitro assays with PtVDL2 using violaxanthin or diadinoxanthin as substrate. Fig. S4. Investigation of other carotenoids than violaxanthin as potential substrates of PtVDL1. Fig. S5. Kinetics of tautomerization of violaxanthin to neoxanthin and of antheraxanthin to deepoxyneoxanthin by PtVDL1. Fig. S6. In vitro activity of PtVDL1 or PtVDE with and without addition of ascorbate. Fig. S7. Pigment composition of chromalveolate algae for which VDL proteins were functionally characterized. Table S1. Pigment stoichiometries in *N. oceanica* wild type, the *vdl* mutant, and two strains of the *vdl* mutant complemented with the native *VDL* gene (*vdl* + *VDL*). Table S2. Pigment stoichiometries in leaves from *N. benthamiana* transiently expressing PtVDL1 fused either to transit peptide tp_NtVDE_ for luminal targeting or to tp_AtZEP_ for stromal targeting and in leaves expressing PtVDL2 fused with tp_NtVDE_ for luminal targeting. Table S3. Pigment stoichiometries in leaves from *N. benthamiana* transiently expressing either VDL from algae with diadinoxanthin cycle or VDL from algae with violaxanthin cycle. Data file S1. Results of targeting prediction for VDL and VDE proteins. Data file S2. Algal sources and database accessions of VDE family protein sequences analyzed in this work. Data file S3. PCR templates and primers used for generation of expression constructs used in this work. Data file S4. Strain-specific single nucleotide polymorphisms in the genes amplified in this work.

## Appendix

View/request a protocol for this paper from *Bio-protocol*.
