# Supplementary material for: An algal enzyme required for biosynthesis of the most abundant marine carotenoids
Source: Sci Adv. 2020 Mar 4;6(10):eaaw9183. doi: 10.1126/sciadv.aaw9183 (PMC7056318; doi:10.1126/sciadv.aaw9183)
Supplement: http://advances.sciencemag.org/cgi/content/full/6/10/eaaw9183/DC1 [file supp_6_10_eaaw9183__index.html]

Science Advances | Science AdvancesAAASSearchScience AdvancesMenu

## Supplementary Materials

**The PDF file includes:**

- Fig. S1. Tentative identification of latoxanthin from *N. oceanica*.
- Fig. S2. Midpoint-rooted maximum likelihood tree of VDE family proteins from selected species of chromalveolate algae and Viridiplantae (land plants and green algae).
- Fig. S3. In vitro assays with PtVDL2 using violaxanthin or diadinoxanthin as substrate.
- Fig. S4. Investigation of other carotenoids than violaxanthin as potential substrates of PtVDL1.
- Fig. S5. Kinetics of tautomerization of violaxanthin to neoxanthin and of antheraxanthin to deepoxyneoxanthin by PtVDL1.
- Fig. S6. In vitro activity of PtVDL1 or PtVDE with and without addition of ascorbate.
- Fig. S7. Pigment composition of chromalveolate algae for which VDL proteins were functionally characterized.
- Table S1. Pigment stoichiometries in *N. oceanica* wild type, the *vdl* mutant, and two strains of the *vdl* mutant complemented with the native *VDL* gene (*vdl* + *VDL*).
- Table S2. Pigment stoichiometries in leaves from *N. benthamiana* transiently expressing PtVDL1 fused either to transit peptide tpNtVDE for luminal targeting or to tpAtZEP for stromal targeting and in leaves expressing PtVDL2 fused with tpNtVDE for luminal targeting.
- Table S3. Pigment stoichiometries in leaves from *N. benthamiana* transiently expressing either VDL from algae with diadinoxanthin cycle or VDL from algae with violaxanthin cycle.

Download PDF

**Other Supplementary Material for this manuscript includes the following:**

- Data file S1 (Microsoft Excel format). Results of targeting prediction for VDL and VDE proteins.
- Data file S2 (Microsoft Excel format). Algal sources and database accessions of VDE family protein sequences analyzed in this work.
- Data file S3 (Microsoft Excel format). PCR templates and primers used for generation of expression constructs used in this work.
- Data file S4 (Microsoft Excel format). Strain-specific single nucleotide polymorphisms in the genes amplified in this work.

**Files in this Data Supplement:**

- Adobe PDF - aaw9183\_SM.pdf
