# Supplementary material for: An algal enzyme required for biosynthesis of the most abundant marine carotenoids
Source: Sci Adv. 2020 Mar 4;6(10):eaaw9183. doi: 10.1126/sciadv.aaw9183 (PMC7056318; doi:10.1126/sciadv.aaw9183)
Supplement: Download PDF [file aaw9183_SM.pdf]

Science Advances
AAAS

advances.sciencemag.org/cgi/content/full/6/10/eaaw9183/DC1

Supplementary Materials for

An algal enzyme required for biosynthesis of the most abundant marine carotenoids

O. Dautermann, D. Lyska, J. Andersen-Ranberg, M. Becker, J. Fröhlich-Nowoisky, H. Gartmann, L. C. Krämer, K. Mayr, D. Pieper, L. M. Rij, H. M.-L. Wipf, K. K. Niyogi, M. Lohr*

*Corresponding author. Email: lohr@uni-mainz.de

Published 4 March 2020, *Sci. Adv.* **6**, eaaw9183 (2020)
DOI: 10.1126/sciadv.aaw9183

The PDF file includes:

Fig. S1. Tentative identification of latoxanthin from *N. oceanica*.
Fig. S2. Midpoint-rooted maximum likelihood tree of VDE family proteins from selected species of chromalveolate algae and Viridiplantae (land plants and green algae).
Fig. S3. In vitro assays with PtVDL2 using violaxanthin or diadinoxanthin as substrate.
Fig. S4. Investigation of other carotenoids than violaxanthin as potential substrates of PtVDL1.
Fig. S5. Kinetics of tautomerization of violaxanthin to neoxanthin and of antheraxanthin to deepoxyneoxanthin by PtVDL1.
Fig. S6. In vitro activity of PtVDL1 or PtVDE with and without addition of ascorbate.
Fig. S7. Pigment composition of chromalveolate algae for which VDL proteins were functionally characterized.
Table S1. Pigment stoichiometries in *N. oceanica* wild type, the *vdl* mutant, and two strains of the *vdl* mutant complemented with the native *VDL* gene (*vdl* + *VDL*).
Table S2. Pigment stoichiometries in leaves from *N. benthamiana* transiently expressing PtVDL1 fused either to transit peptide tp_{NtVDE} for luminal targeting or to tp_{AtZEP} for stromal targeting and in leaves expressing PtVDL2 fused with tp_{NtVDE} for luminal targeting.
Table S3. Pigment stoichiometries in leaves from *N. benthamiana* transiently expressing either VDL from algae with diadinoxanthin cycle or VDL from algae with violaxanthin cycle.

Other Supplementary Material for this manuscript includes the following:

(available at advances.sciencemag.org/cgi/content/full/6/10/eaaw9183/DC1)

Data file S1 (Microsoft Excel format). Results of targeting prediction for VDL and VDE proteins.
Data file S2 (Microsoft Excel format). Algal sources and database accessions of VDE family protein sequences analyzed in this work.

Data file S3 (Microsoft Excel format). PCR templates and primers used for generation of expression constructs used in this work.
Data file S4 (Microsoft Excel format). Strain-specific single nucleotide polymorphisms in the genes amplified in this work.

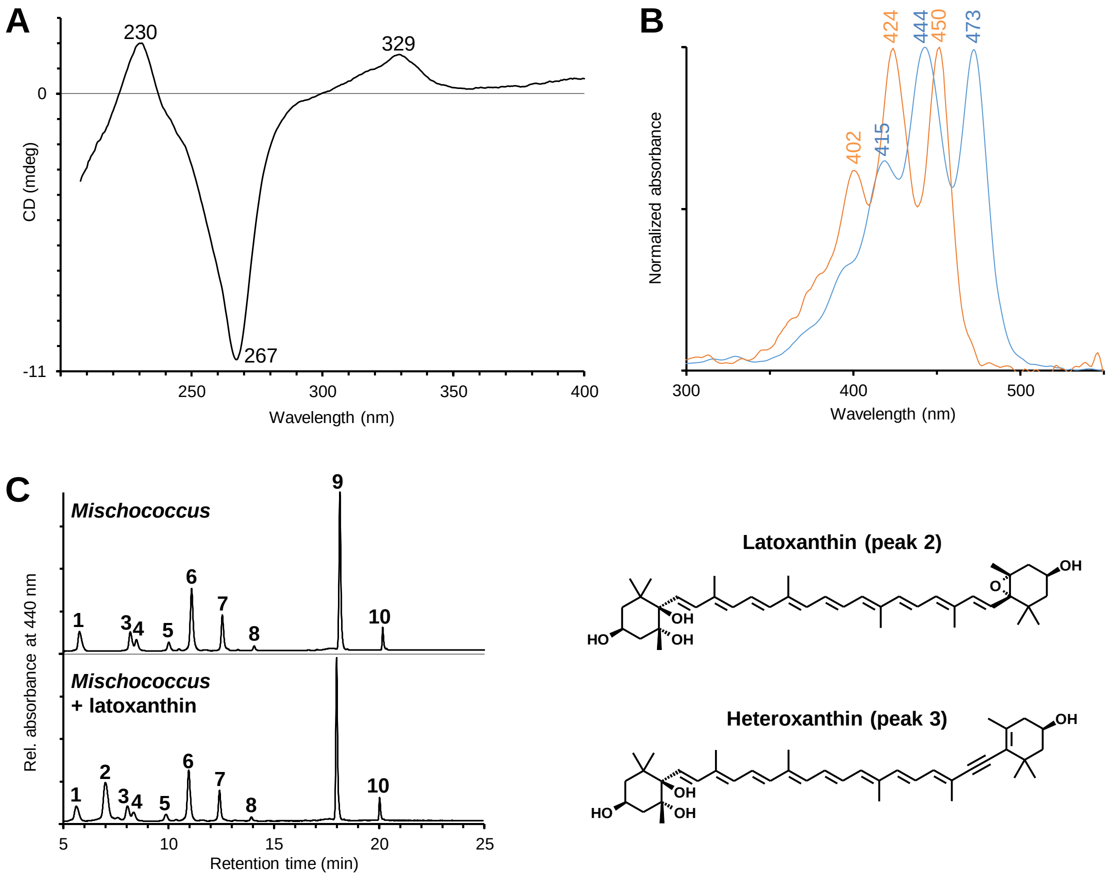

Fig. S1. Tentative identification of latoxanthin from *N. oceanica*. (**A**) CD spectrum of the isolated pigment (230 µM in ethanol). (**B**) UV/VIS absorbance spectrum of the pigment before (blue) and after (orange) treatment with dilute HCl that results in a 22 nm hypsochromic shift of the absorbance maxima diagnostic for the presence of a 5,6-epoxy group. (**C**) Spiking of a pigment extract from the xanthophyte alga *Mischococcus sphaerocephalus* with latoxanthin from *N. oceanica* and analysis on HPLC system IIb confirms a shorter retention time of latoxanthin (peak 2) in comparison to the structurally similar but less polar heteroxanthin (3). Other peaks represent chlorophyll c_1+c_2 (1), neoxanthin (4), dinoxanthin (5), diadinoxanthin (6), vaucheriaxanthin acyl esters (7, 8), chlorophyll *a* (9) and β-carotene (10). The experimental details are described in Materials and Methods.

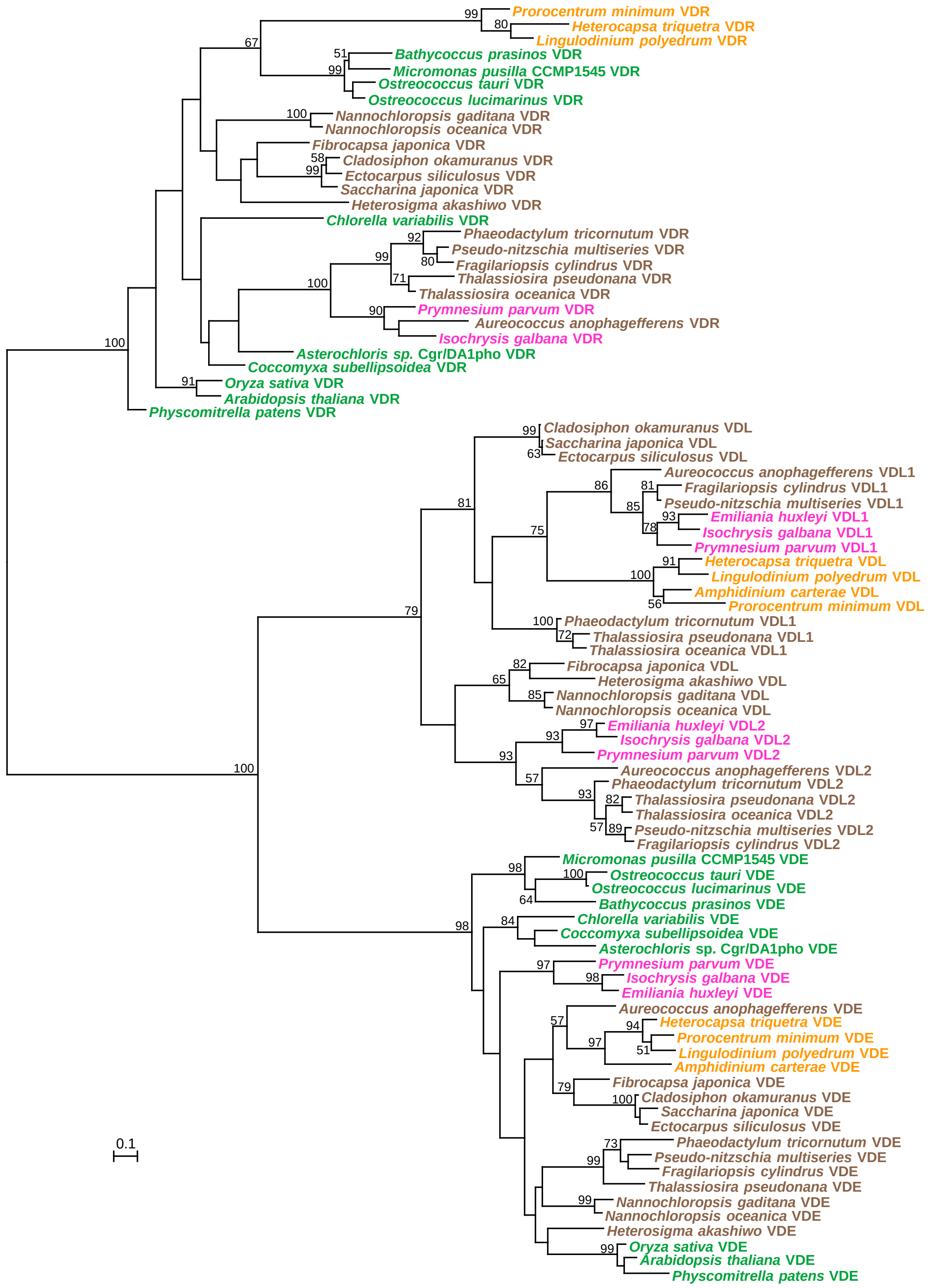

Fig. S2. Midpoint-rooted maximum likelihood tree of VDE family proteins from selected species of chromalveolate algae and Viridiplantae (land plants and green algae). The tree was inferred from a protein sequence alignment containing 142 distinct patterns. Results of rapid bootstrap analysis with RAxML (100 replicates) are shown for values above 50%. Branch lengths are proportional to the number of substitutions per site (see scale bar). Species names are color-coded according to their taxonomic affiliation: Viridiplantae in green, Heterokontophyta in brown, Dinophyta in orange, and Haptophyta in magenta. For sequence accessions, see data S2.

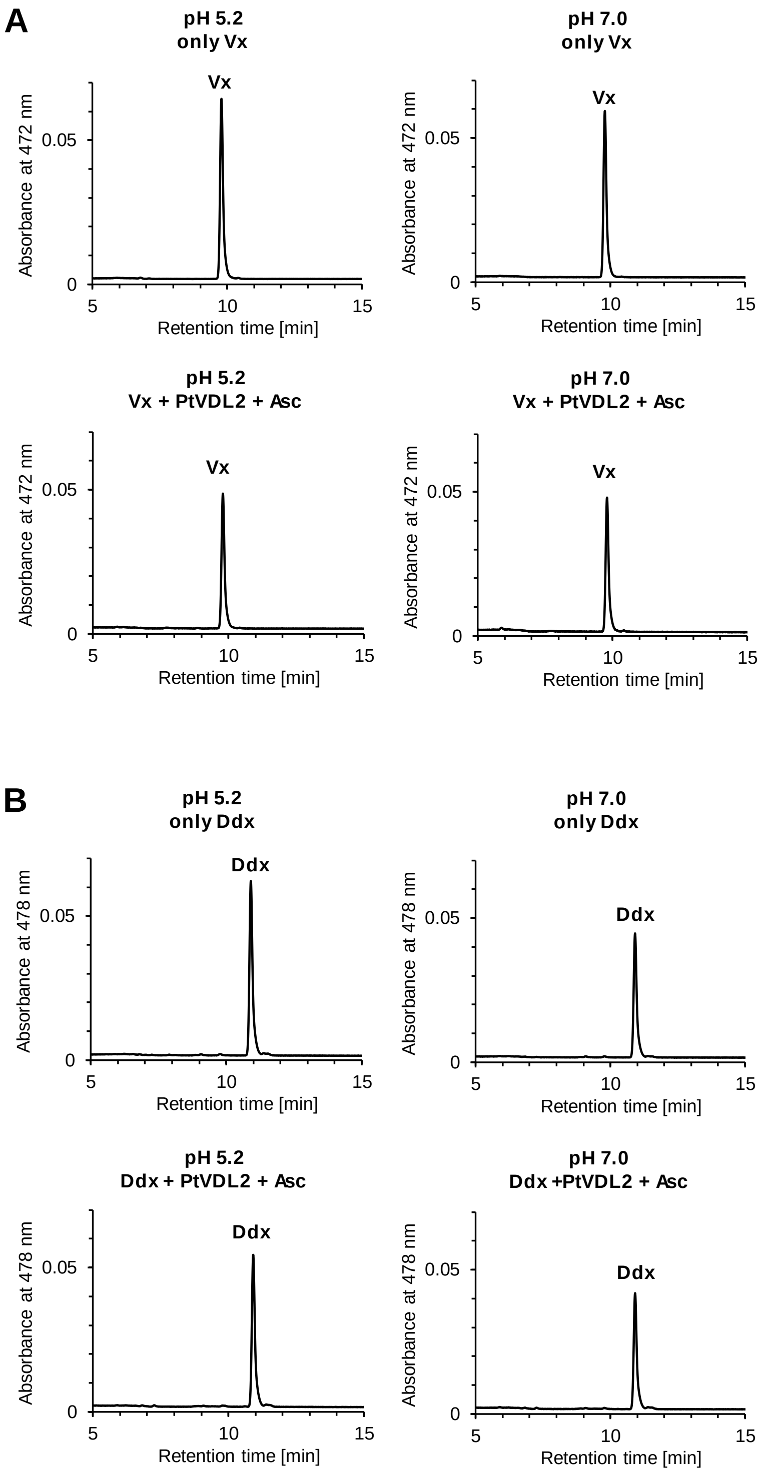

Fig. S3. In vitro assays with PtVDL2 using violaxanthin or diadinoxanthin as substrate. HPLC analyses (system II) of samples from *in vitro* assays with recombinant PtVDL2 at pH 5.2 or pH 7.0 and incubation times of 3 h. The assays contained either (**A**) violaxanthin (Vx) or (**B**) diadinoxanthin (Ddx) as substrate and recombinant PtVDL2 together with 30 mM ascorbate (Asc) (lower chromatograms in both panels). Assays without addition of PtVDL2 and ascorbate served as controls (upper chromatograms in both panels).

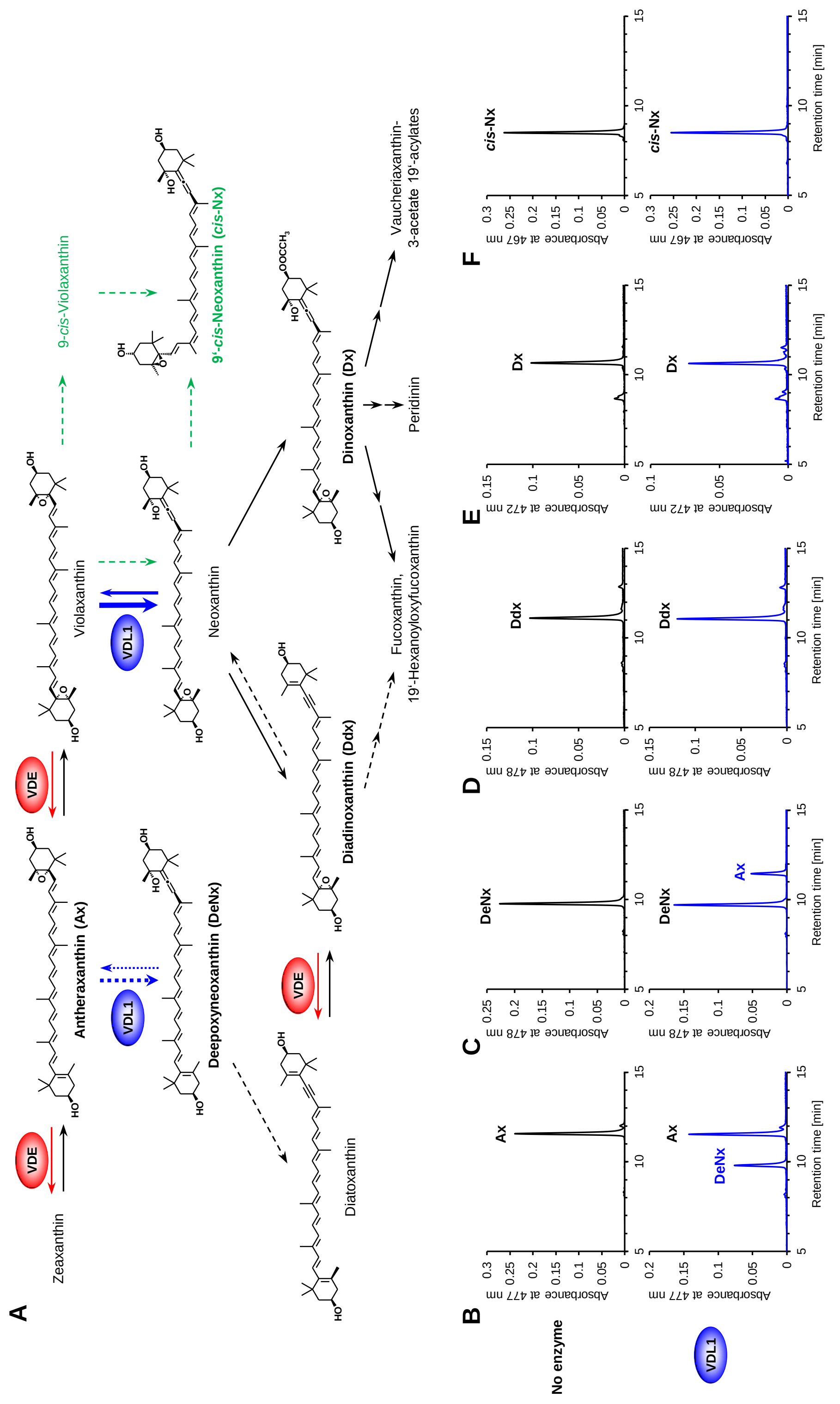

Fig. S4. Investigation of other carotenoids than violaxanthin as potential substrates of PtVDL1. (**A**) Proposed biosynthetic relations of carotenoids tested (substrate names in bold; other features as in Fig. 4). (**B-F**) HPLC analyses (system II) of pigment extracts from *in vitro* assays after 24 h incubation of different carotenoids with or without PtVDL1 show: (**B-C**), tautomerisation of antheraxanthin and deepoxyneoxanthin (reaction products labelled blue); and (**D-E**), no activity with diadinoxanthin and dinoxanthin, two epoxy carotenoids synthesized from neoxanthin. (**F**) 9'-*cis*-neoxanthin that is present in land plants and green algae but not in chromalveolates, was also not a substrate for PtVDL1. The experiment was repeated once with similar results using a different VDL1 preparation. Other experimental details are described in Materials and Methods.

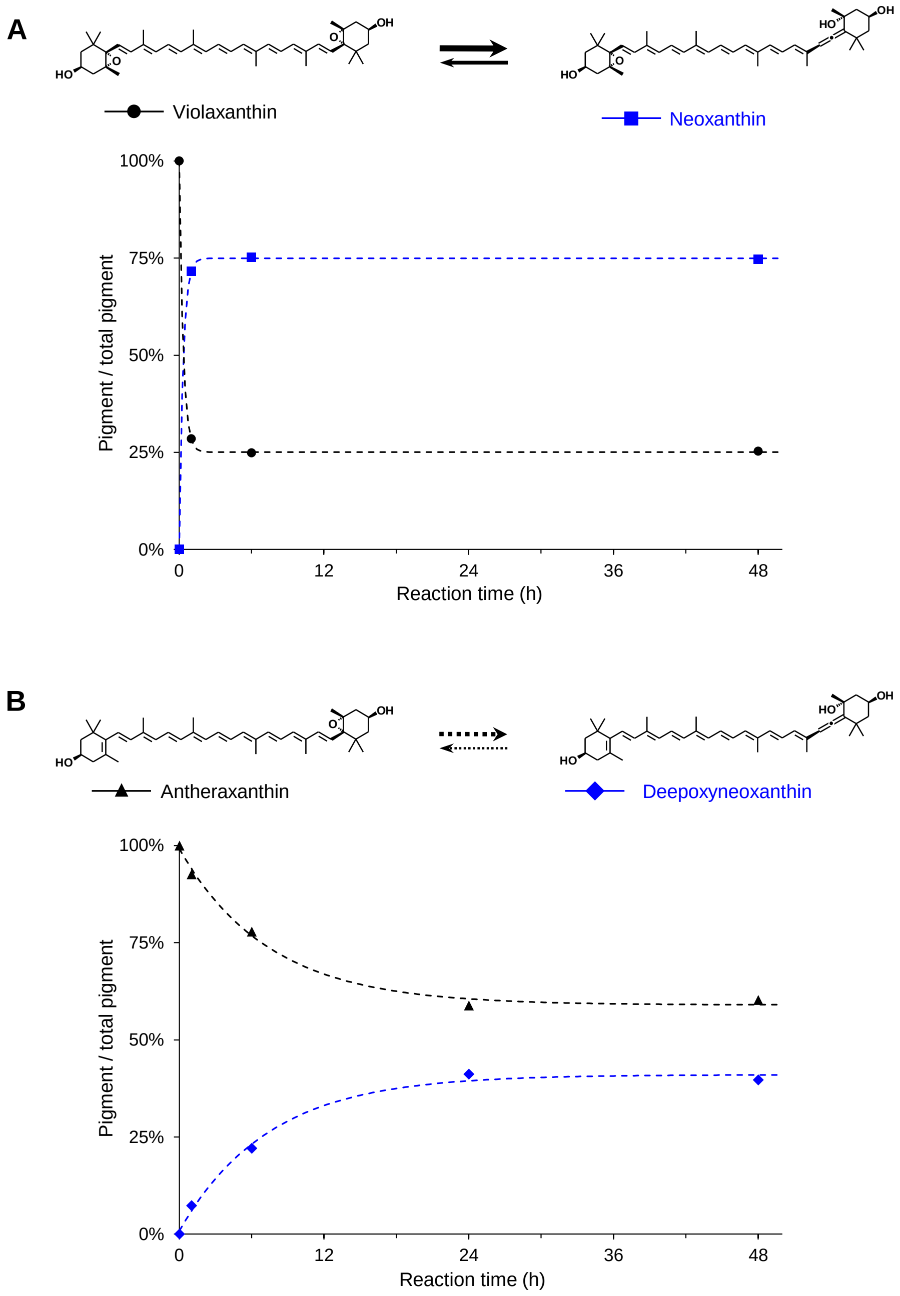

Fig. S5. Kinetics of tautomerization of violaxanthin to neoxanthin and of antheraxanthin to deepoxyneoxanthin by PtVDL1. (**A**) *In vitro* kinetics of tautomerization of violaxanthin to neoxanthin showing almost complete equilibration of the reaction after 1h. (**B**) Under identical reaction conditions and using the same VDL preparation, tautomerization of antheraxanthin to deepoxyneoxanthin was much slower. The experiment was done twice with similar results using two different VDL preparations. Other experimental details are described in Materials and Methods.

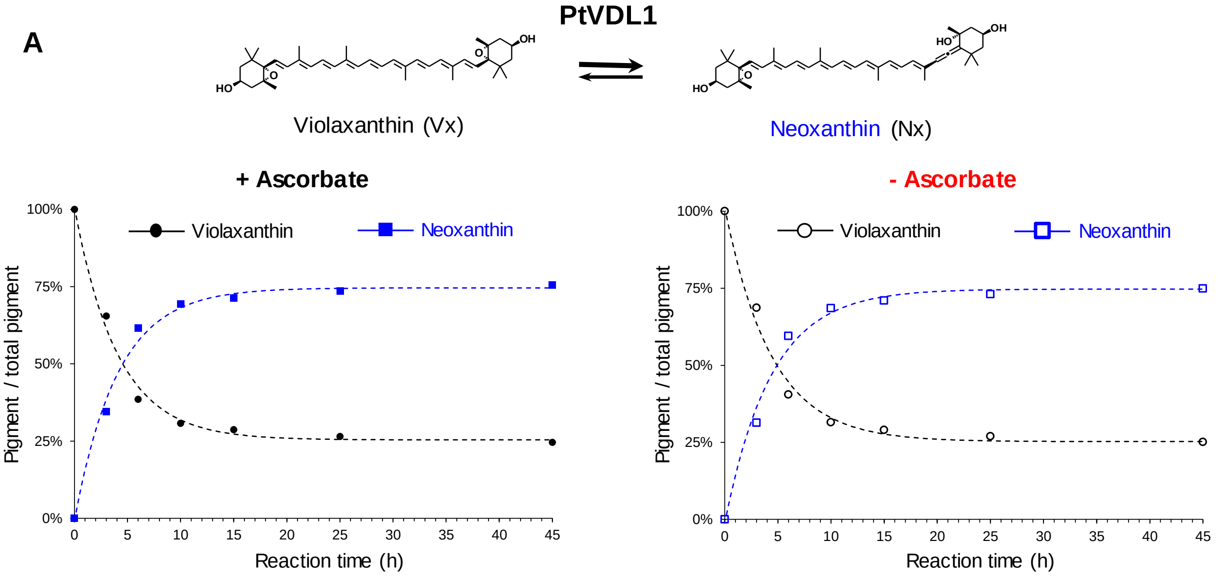

$$[Vx]_{(t)} = [Vx]_{(equil.)} + [Nx]_{(equil.)} \cdot e^{(k \cdot t)}$$

with $[Vx]_{(equil.)} = 0.25$

and $[Nx]_{(equil.)} = 0.75$

Exp.#	$k_{(+Asc)}$	$k_{(-Asc)}$	$k_{(-Asc)} / k_{(+Asc)}$ (%)
1	0.246	0.228	93
2	0.431	0.387	90
3	0.134	0.118	88

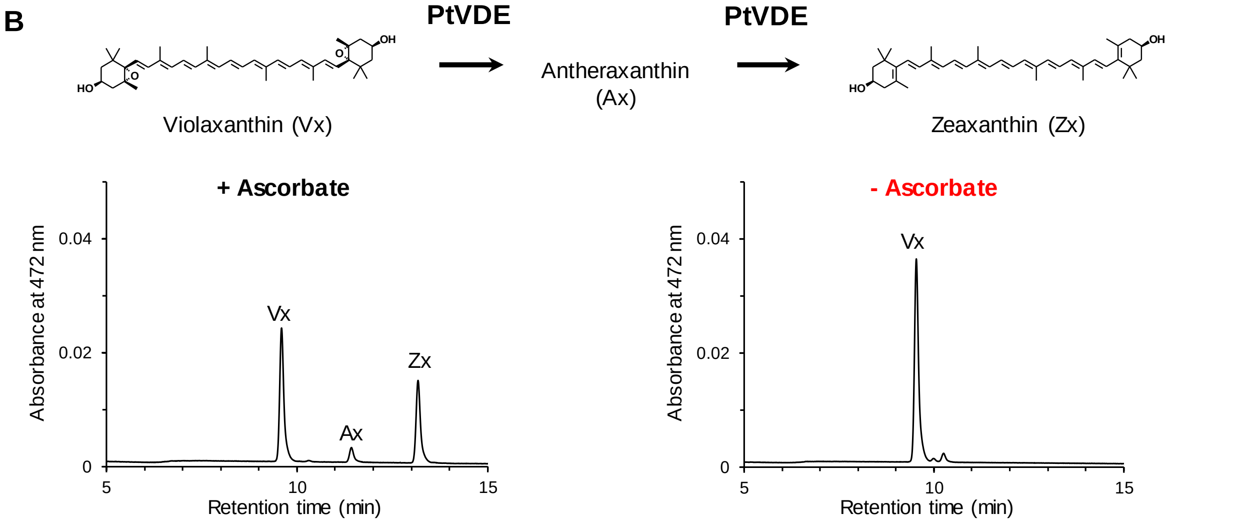

Fig. S6. In vitro activity of PtVDL1 or PtVDE with and without addition of ascorbate. (**A**) *In vitro* kinetics of tautomerization of violaxanthin to neoxanthin by recombinant PtVDL1 with 30 mM ascorbate (left) or without ascorbate (right) present in the assay. Data were fitted using the equilibrium concentration function given below the kinetic plots. The table compares the rate constants k from three experiments, demonstrating a reduction of the reaction rates by about 10% when ascorbate was omitted. (**B**) HPLC chromatograms (system II) of samples from *in vitro* assays after 10 min incubation of recombinant PtVDE with violaxanthin as substrate and either 30 mM ascorbate (left) or no ascorbate (right) present in the assay. The experiments were done three times with similar results using different preparations of PtVDL1 and PtVDE, respectively. Other experimental details are described in Materials and Methods.

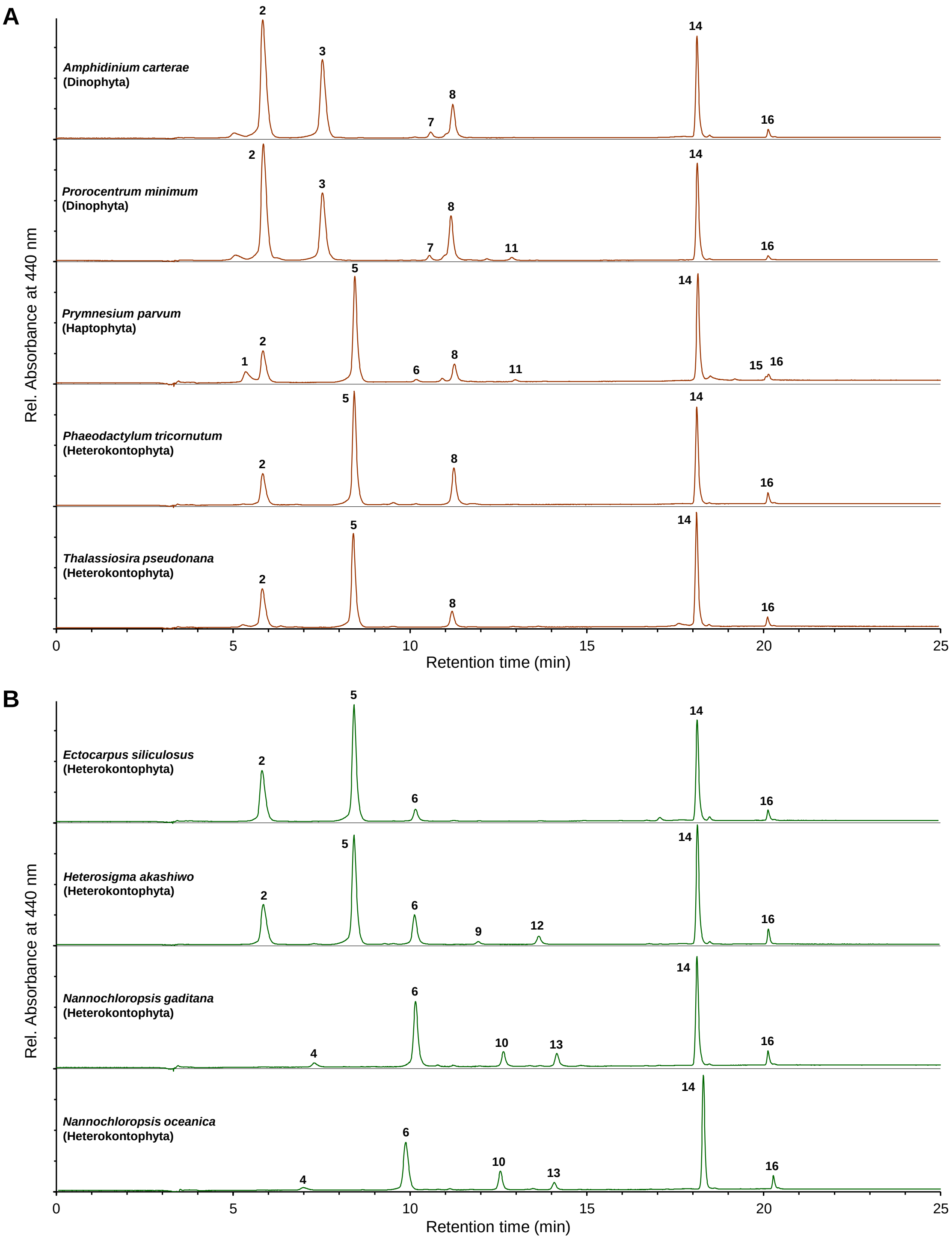

Fig. S7. Pigment composition of chromalveolate algae for which VDL proteins were functionally characterized. (**A**) HPLC chromatograms (system IIb) of pigment extracts from algae that use the diadinoxanthin cycle for photoprotection, confirming the presence of diadinoxanthin (peak 8) while violaxanthin (peak 6) is barely detectable. (**B**) Chromatograms (system IIb) of pigment extracts from algae that use the violaxanthin cycle, showing the presence of substantial amounts of violaxanthin (6). Other labeled peaks were identified as chlorophyll c_3 (1), chlorophyll c_1+c_2 (2), peridinin (3), latoxanthin (4), fucoxanthin (5), dinoxanthin (7), antheraxanthin (9), diatoxanthin (11), zeaxanthin (12), vaucheriaxanthin acyl esters (10, 13), chlorophyll *a* (14), α-carotene (15), and β-carotene (16). The experimental details are described in Materials and Methods.

Table S1. Pigment stoichiometries in *N. oceanica* wild type, the *vdl* mutant, and two strains of the *vdl* mutant complemented with the native *VDL* gene (*vdl* + *VDL*).

N. oceanica [sample size]	Latoxanthin (L)	Violaxanthin (V)	Antheraxan. (A)	Zeaxanthin (Z)	V+A+Z	Vaux I	Vaux II	V+A+Z+L+ Vaux I+II	β-carotene	9-*cis*-β-carotene
Wild type [4]	25 ± 4	302 ± 16	2 ± 0	7 ± 1	310 ± 18	96 ± 5	34 ± 5	465 ± 23	57 ± 7	2 ± 2
vdl mutant [4]	36 ± 4	439 ± 33	6 ± 2	14 ± 3	459 ± 29	0	0	495 ± 31	63 ± 8	2 ± 2
p values < 0.05	0.007	0.0003	0.002	0.006	0.0001	$1*10^{-8}$	$8*10^{-6}$			
vdl+*VDL* #2 [2]	24 ± 3	270 ± 17	3 ± 0	23 ± 5	294 ± 24	127 ± 9	36 ± 7	480 ± 23	62 ± 9	2 ± 3
p values < 0.05				0.003		0.004				
vdl+*VDL* #4 [4]	19 ± 3	256 ± 4	3 ± 4	17 ± 8	276 ± 13	91 ± 9	49 ± 4	435 ± 21	48 ± 1	1 ± 1
p values < 0.05		0.002		0.0498	0.02		0.003		0.046	

Pigment data are expressed as mmol / mol Chl *a*, giving arithmetic means ± s.d. of biological replicates. P values for pigment stoichiometries differing significantly (P < 0.05) between wild type and the respective mutants were calculated using two-tailed Student's *t*-test statistics. Samples were analyzed using HPLC system IIb. Other experimental details are described in Methods.

Table S2. Pigment stoichiometries in leaves from *N. benthamiana* transiently expressing PtVDL1 fused either to transit peptide tp_{NtVDE} for luminal targeting or to tp_{AtZEP} for stromal targeting and in leaves expressing PtVDL2 fused with tp_{NtVDE} for luminal targeting.

Protein [sample size]	Chl *b*	Lutein	Violaxan. (V)	Anthera-xan. (A)	Zeaxan. (Z)	V+A+Z	*trans*-Neoxanthin	9'-*cis*-Neoxan.	Deepoxy-neoxan.	β-Carotene	*9-cis*-β-Carotene
Non-infiltrated control [8]	293 ± 15	164 ± 8	56 ± 5	8 ± 2	2 ± 2	66 ± 6	0	43 ± 4	0	66 ± 10	5 ± 0
tp_{NtVDE}-PtVDL1 [10]	314 ± 13	198 ± 15	38 ± 5	6 ± 4	3 ± 5	46 ± 7	14 ± 5	69 ± 8	2 ± 2	72 ± 7	6 ± 1
p values < 0.05	0.006	$4*10^{-5}$	$5*10^{-7}$			$1*10^{-5}$		$2*10^{-7}$			
tp_{AtZEP}-PtVDL1 [6]	316 ± 15	208 ± 10	44 ± 18	13 ± 6	11 ± 12	67 ± 6	2 ± 2	59 ± 2	0	73 ± 19	6 ± 1
p values < 0.05	0.02	$1*10^{-6}$						$3*10^{-6}$			
tp_{NtVDE}-PtVDL2 [7]	298 ± 7	178 ± 4	50 ± 7	8 ± 3	4 ± 2	63 ± 4	0	52 ± 2	0	83 ± 7	6 ± 0
p values < 0.05		0.002						0.0002		0.001	0.01

Pigment data are expressed as mmol / mol Chl *a*, giving arithmetic means ± s.d. of biological replicates. P values for pigment stoichiometries differing significantly (P < 0.05) between control leaves and leaves expressing the respective VDL proteins were calculated using two-tailed Student's *t*-test statistics. Samples were analyzed using HPLC system I. Other experimental details are described in Methods.

Table S3. Pigment stoichiometries in leaves from *N. benthamiana* transiently expressing either VDL from algae with diadinoxanthin cycle or VDL from algae with violaxanthin cycle.

Source of VDL [sample size]	Chl *b*	Lutein	Violaxan. (V)	Anthera-xan. (A)	Zeaxan. (Z)	V+A+Z	*trans*-Neoxan.	9'-*cis*-Neoxan.	Deepoxy-neoxan.	β-Carotene	*9-cis*-β-Carotene
Algae with diadinoxanthin cycle											
Amphidinium carterae [3]	311 ± 14	188 ± 3	30 ± 2	8 ± 1	6 ± 2	45 ± 3	16 ± 1	65 ± 2	3 ± 1	86 ± 5	6 ± 1
Prorocentrum minimum [3]	307 ± 2	187 ± 2	34 ± 2	6 ± 0	3 ± 1	43 ± 2	15 ± 2	62 ± 2	2 ± 0	81 ± 3	6 ± 0
Prymnesium parvum [3]	305 ± 7	182 ± 7	32 ± 1	6 ± 1	3 ± 1	41 ± 2	14 ± 2	64 ± 2	2 ± 1	89 ± 4	6 ± 0
Phaeodactylum tricornutum [5]	303 ± 2	183 ± 2	31 ± 4	9 ± 6	10 ± 10	50 ± 13	10 ± 3	62 ± 1	3 ± 1	77 ± 9	6 ± 0
Thalassiosira pseudonana [3]	308 ± 4	177 ± 5	26 ± 1	13 ± 2	11 ± 4	50 ± 5	11 ± 2	62 ± 6	5 ± 1	84 ± 3	6 ± 0
Average [17]	304 ± 7	183 ± 5	31 ± 3	9 ± 4	7 ± 6	46 ± 8	13 ± 3	63 ± 3	3 ± 1	83 ± 7	6 ± 0
Algae with violaxanthin cycle											
Ectocarpus siliculosus [3]	307 ± 6	180 ± 5	33 ± 3	13 ± 2	10 ± 4	56 ± 5	5 ± 1	60 ± 1	3 ± 2	81 ± 4	6 ± 1
Hetersosigma akashiwo [3]	320 ± 1	186 ± 3	36 ± 2	7 ± 0	6 ± 0	50 ± 3	6 ± 0	59 ± 1	2 ± 0	85 ± 1	6 ± 1
Nannochloropsis gaditana [3]	308 ± 7	197 ± 8	42 ± 6	13 ± 1	9 ± 1	64 ± 6	4 ± 1	59 ± 4	4 ± 0	64 ± 7	7 ± 0
Nannochloropsis oceanica [5]	303 ± 3	180 ± 4	38 ± 5	10 ± 3	7 ± 4	55 ± 2	4 ± 1	59 ± 2	3 ± 1	81 ± 10	7 ± 0
Average [14]	309 ± 8	185 ± 8	37 ± 5	11 ± 3	8 ± 3	56 ± 6	4 ± 1	59 ± 2	3 ± 1	78 ± 10	7 ± 1
p values < 0.05			$1*10^{-4}$			$7*10^{-4}$	$1*10^{-10}$	$4*10^{-4}$			

Pigment data are expressed as mmol / mol Chl *a*, giving arithmetic means ± s.d. of biological replicates. P values for pigment stoichiometries differing significantly ($P < 0.05$) between leaves expressing VDL from algae with diadinoxanthin cycle and leaves expressing VDL from algae with violaxanthin cycle were calculated using two-tailed Student's *t*-test statistics. Samples were analyzed using HPLC system Ib. Other experimental details are described in Methods.

Captions for Supplementary Data

Data file S1. Results of targeting prediction for VDL and VDE proteins.

Data file S2. Algal sources and database accessions of VDE family protein sequences analyzed in this work.

Data file S3. PCR templates and primers used for generation of expression constructs used in this work.

Data file S4. Strain-specific single nucleotide polymorphisms in the genes amplified in this work.
